# Physiological and Ecological Responses of the Bloom-Forming Diatom *Achnanthidium catenatum* to Nutrients

**DOI:** 10.3390/plants15081229

**Published:** 2026-04-16

**Authors:** Tian-Yu Yang, Li-Gen Tang, Ya-Ran Yun, Yi-Lin Bai, Guo-Feng Pei

**Affiliations:** Hubei Provincial Key Laboratory for Protection and Application of Special Plant Germplasm in Wuling Area of China, South-Central Minzu University, Wuhan 430074, China; 2024120522@mail.scuec.edu.cn (T.-Y.Y.); 202221141283@mail.scuec.edu.cn (L.-G.T.); 202421141252@mail.scuec.edu.cn (Y.-R.Y.); 2025110335@mail.scuec.edu.cn (Y.-L.B.)

**Keywords:** *Achnanthidium catenatum*, nitrogen, phosphorus, peak density, extracellular polymeric substances, multicellular chain colonies

## Abstract

*Achnanthidium catenatum* exhibits both epiphytic and planktonic ecological types and forms multiple large-scale diatom blooms in different drinking water reservoirs. This study determined its growth and physiological characteristics under different nitrogen (N) or phosphorus (P) conditions. The study found that N ≤ 2.5 mg/L promoted its growth, while the promoting effect weakened at ≥5 mg/L. The lag phase of the growth cycle was shorter, taking only 6 days to reach peak density; meanwhile, it showed strong adaptability to P (0.5–3.5 mg/L), with peak density occurring by approximately 12 days. It was found that N-induced blooms formed earlier and lasted longer, whereas P-induced blooms were relatively delayed, more intense, and shorter in duration. Low and high concentrations of N, as well as P concentrations (≥0.1 mg/L), significantly promoted the formation of multicellular chain colonies (*p* < 0.05). The percentage of chain colonies was relatively higher during the lag phase and tended to exist as single cells during the stationary phase, at which time the colloidal extracellular polymeric substance (CEPS) content was higher and significantly correlated with changes in cell density. Alkaline phosphatase activity (APA) and malondialdehyde (MDA) content varied markedly under different N or P concentrations (*p* < 0.05). These results reveal the potential impact of N or P variations on the bloom-forming capacity of *A. catenatum*.

## 1. Introduction

There are many freshwater diatom species that cause algal blooms. Their physiological and ecological characteristics vary, and the most common dominant bloom-forming diatoms include *Stephanodiscus* and *Cyclotella* [1,2], planktonic diatoms whose explosive growth mainly occurs in rivers [3,4]. In contrast, among the genus *Achnanthidium*, which typically attaches to substrates via mucilage stalks [5], *Achnanthidium catenatum* is the only species that exhibits both epiphytic and planktonic lifestyles. When planktonic, it exists as individual or multicellular chain colonies, and can form large-scale blooms in lakes and reservoirs with relatively low nutrient levels [6,7,8]. In the spring of 2010, large-scale diatom blooms occurred in several drinking water source reservoirs in Ningbo, Taizhou, and Shaoxing, Zhejiang Province [6]. During the bloom period, the cell density of *A*. *catenatum* exceeded 1 × 10^8^ cells/L, water transparency decreased, some physical and chemical indicators exceeded the standard, and water quality deteriorated; these factors affect the safe utilization of water sources [6] and have aroused widespread attention. It has been reported that blooms of *A. catenatum* frequently occur in lakes and reservoirs [9,10,11]. Previously published studies have reported that blooms of *A. catenatum* mostly occur after rainfall and rising temperatures during the rainy season, but their scale and duration differ significantly [6]. During blooms, water temperature fluctuates widely (12.6–25.7 °C), and concentrations of N and P are relatively low; moreover, both water temperature and nutrient concentrations differ greatly among different water bodies [9,10,11]. For example, the variation ranges of total nitrogen and total phosphorus were 0.2–2.07 mg/L and 0.01–0.03 mg/L, respectively. However, studies on the effects of nutrients during bloom formation remain insufficient and have failed to identify the N and P thresholds responsible for bloom formation, intensity, and duration.

Diatom bloom formation is driven by the interplay of multiple factors, including temperature, light, nutrients, and climatic conditions, with N and P nutrient loads among the primary drivers. Lyu et al. [12] studied the correlation between water nutrient concentrations and diatom blooms in Hengshan Reservoir (Taihu Lake Basin), and their findings showed that N and P concentrations significantly affect the population density and abundance of diatoms. Similarly, Wu et al. [3] reported a significantly positive correlation between diatom bloom occurrence in the middle and lower reaches of the Hanjiang River and silicon (Si) and N concentrations. Further research on dominant bloom-forming diatoms has shown that the optimal N and P concentration range for *Stephanodiscus* sp. (a dominant bloom-forming diatom in the Hanjiang River) overlapped with nutrient levels during the bloom period [13]. Yang et al. [14] and Yu et al. [15] also showed that N, P, and Si concentrations significantly affect the growth, reproduction, and physiological metabolism of *Navicula patrickae*, *Nitzschia panduriformis*, and *Cyclotella cryptica*. Extracellular polymeric substances (EPSs) primarily exist in two forms: attached (AEPSs) and colloidal (CEPSs). AEPSs have a crucial effect in diatom migration, movement, habitat stabilization, and colonial formation [16]. Previous studies have confirmed that chain-forming diatoms can adhere to one another by secreting EPSs, which promotes the formation of multicellular chain colonies [17]. Many studies have shown the content of diatom EPSs is closely linked to their growth cycle, and different nutrients have distinct effects on secretion dynamics [18,19].

Nutrient loading is a major limiting factor for the growth of *A. catenatum* [6]. Even though N and P concentrations in water bodies are relatively low during bloom outbreaks [6], it is hypothesized that pulsed nutrient loading caused by rainfall may be one of the main factors for its blooms in lakes and reservoirs. However, research on how N and P influence EPS production, multicellular chain colony formation, and the occurrence and decline of blooms is limited. Therefore, this study uses a purified strain of *A. catenatum* isolated from Dongqian Lake (Ningbo, Zhejiang Province) during its algal bloom period as the experimental material, and determines its growth and physiological characteristics under different N and P gradients. The study aimed to: (1) explore *A. catenatum*’s biological traits in response to N and P; (2) clarify the effects of varying nutrient concentrations on its growth and physiology; and (3) estimate the N and P thresholds for bloom formation. The findings provide a scientific basis for *A. catenatum* bloom early warning and control.

## 2. Results

### 2.1. Effects of N on the Growth and Colony Formation of Achnanthidium catenatum

**Figure 1:**
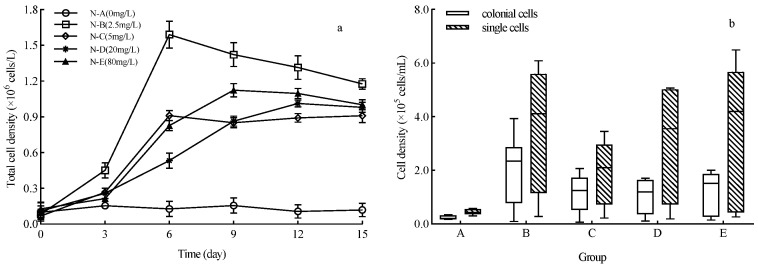
Growth characteristics of *Achnanthidium catenatum* in water bodies with different exogenous nitrogen (N) concentrations. (**a**) Dynamic changes in the total cell density of *Achnanthidium catenatum* across different N treatment groups over the 15-day cultivation period; (**b**) density distribution of colonial cells (represented by solid bars) and single cells (represented by hatched bars) in each N treatment group at the end of cultivation.

In group N-B, *A. catenatum* had a short lag phase, with total cell density peaking at 1.59 × 10^6^ cells/L on the 6th day and the shortest generation time among all treatments. The diatoms in groups N-D and N-E entered the stationary phase on the 9th day (Figure 1a), and their growth curves showed an “S-shape” during culture. The biomass in group N-B was significantly higher than in other treatments, indicating that 2.5 mg/L is the optimal N concentration for *A. catenatum* growth. When N concentration exceeded 2.5 mg/L, total cell density was significantly higher than the control, but showed no significant difference among groups N-C, N-D, and N-E. These results suggest that low N concentrations promote *A. catenatum* growth, while the promoting effect weakened at high concentrations. The N gradient experiment predicts that N will induce earlier and longer-lasting *A. catenatum* blooms, but with weaker intensity.

The multicellular chain colony densities of *A. catenatum* were higher in all treatment groups than the control; the proportion of multicellular chain colonies differed significantly from the control under low-concentration B (2.5 mg/L) and high-concentration E (80 mg/L) (*p* < 0.05). Among all treatments, only group B (2.5 mg/L) and group D (20 mg/L) showed significant differences in the proportion of multicellular chain colonies (*p* < 0.05). Within each treatment, the densities of multicellular and single-cell colonies differed significantly (*p* < 0.05). Throughout the growth cycle, single cells and multicellular chain colonies coexisted in all treatments during the first 6 days of the log phase; after cell density peaked in the stationary phase, multicellular chain colonies gradually declined in all treatments and their proportion fell below 20% (Figure 1b). These results indicated that low or high N concentrations promote the formation of multicellular chain colonies in *A. catenatum*, whereas this diatom tends to exist as single cells during the stationary phase.

### 2.2. Effects of P on the Growth and Multicellular Chain Colony Formation of Achnanthidium catenatum

**Figure 2:**
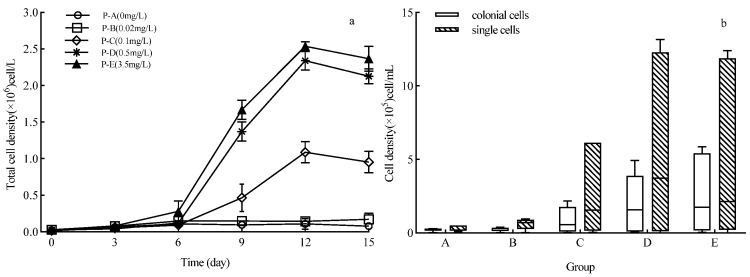
Growth and physiological characteristics of *Achnanthidium catenatum* in water bodies with different exogenous phosphorus (P) concentrations. (**a**) Dynamic changes in the total cell density of *Achnanthidium catenatum* across different P treatment groups over the 15-day cultivation period; (**b**) density distribution of colonial cells (represented by solid bars) and single cells (represented by hatched bars) in each P treatment group at the end of cultivation.

Under different P concentrations, *A. catenatum* maintained a planktonic lifestyle, with no significant difference in total cell density observed in the early culture stage (*p* > 0.05). Total cell density in groups P-C, P-D, and P-E peaked on the 12th day, followed by a rapid decline in the late stationary phase (Figure 2a). This diatom can adapt to a wide range of P concentrations; when P was 0.1–3.5 mg/L, the total cell density was significantly positively correlated with P concentration (*p* < 0.05). Group P-C had significantly lower total cell density than groups P-D and P-E, with no significant difference between the latter two (*p* > 0.05). These results indicate that P has a concentration threshold for promoting *A. catenatum* growth. The P gradient experiment suggests that under such P conditions, algal blooms occur later, last for a shorter period, and have higher intensity.

The density of multicellular chain colonies of *A. catenatum* in all treatment groups was higher than the control. During the first 3 days of growth, the proportion of multicellular chain colonies in each treatment group accounted for 20–30% of the total density, which was significantly higher than the 16–23% recorded in the log phase and stationary phase (*p* < 0.05). For treatment groups with P concentrations ≤ 0.02 mg/L, both multicellular chain colony and single-cell densities remained at low levels throughout the incubation period. In contrast, for treatment groups with P concentrations ≥ 0.1 mg/L, both densities increased with rising P concentration as the culture progressed (Figure 2b). Although multicellular chain colony density showed the same trend as total cell density, single-cell density was significantly higher during the stationary phase, and this dominance became more pronounced with increasing P concentration. These results indicated that P significantly promoted the formation of multicellular chain colonies in *A. catenatum* (*p* < 0.05), and the diatom was dominated by single cells in the stationary phase.

### 2.3. Effects of N and P on EPS Secretion by Achnanthidium catenatum

**Figure 3:**
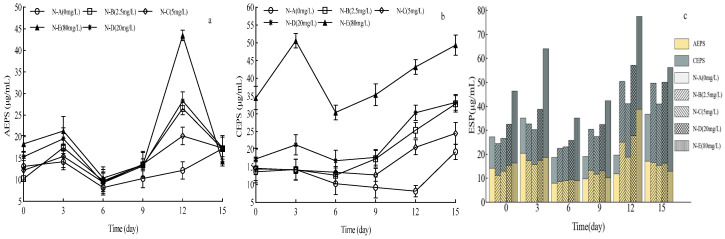
Effects of different N concentrations on extracellular polymeric substance (EPS) content of *Achnanthidium catenatum*. (**a**) Dynamic changes in the attached extracellular polymeric substance (AEPS) content across different N treatment groups during the 15-day cultivation period; (**b**) dynamic changes in the colloidal extracellular polymeric substance (CEPS) content across different N treatment groups over the 15-day cultivation period; (**c**) temporal dynamic changes in the total EPS content across different N treatment groups (throughout the 15-day cultivation period), where the yellow segments represent the AEPS component and the gray segments the CEPS component.

During the experiment, *A. catenatum* in all treatments secreted EPSs during the rapid growth phase, with EPS content peaking in the stationary phase. In the stationary phase, AEPS and CEPS contents increased gradually with rising N concentration in all groups except group N-B. Compared with other treatments, group N-E had significantly higher CEPS than AEPS content (*p* < 0.05) (Figure 3a,b). These results indicate that *A. catenatum* EPS secretion is affected by N concentration: N has a relatively minor effect on AEPS secretion, but CEPS secretion is significantly correlated with N concentration (*p* < 0.05).

**Figure 4:**
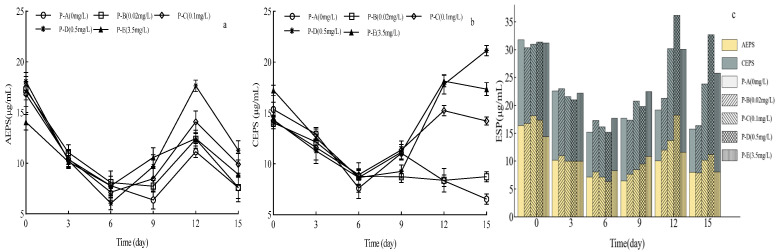
Effects of different P concentrations on EPS content of *Achnanthidium catenatum*. (**a**) Dynamic changes in the attached extracellular polymeric substance (AEPS) content across different P treatment groups during the 15-day cultivation period; (**b**) dynamic changes in the colloidal extracellular polymeric substance (CEPS) content across different P treatment groups over the 15-day cultivation period; (**c**) temporal dynamic changes in the total EPS content across different P treatment groups (throughout the 15-day cultivation period), where the yellow segments represent the AEPS component and the gray segments the CEPS component.

Different P concentrations had distinct effects on *A. catenatum* EPS secretion. During the lag phase, the diatom’s EPS content decreased gradually (Figure 4c). In the late log phase, EPS content peaked synchronously with cell density on the 12th day. Following the lag phase, no significant difference in EPS content existed between groups P-A and P-B (*p* > 0.05). In contrast, groups P-C, P-D, and P-E had significantly higher EPS content than group P-B, and it was significantly positively correlated with P concentration (*p* < 0.05). Significant changes in EPS components occurred during the experiment: AEPS content was slightly higher than CEPS in the lag phase (Figure 4a,b). As the diatoms entered the stationary phase and cell density reached the peak, the EPS content in groups P-C, P-D, and P-E remained significantly higher than group P-B. In high P groups, the CEPS content was significantly higher than AEPS (*p* < 0.05), indicating that CEPS accumulation was closely related to the dominance of unicellular individuals in the stationary phase.

### 2.4. Effects of N and P on APA of Achnanthidium catenatum

**Figure 5:**
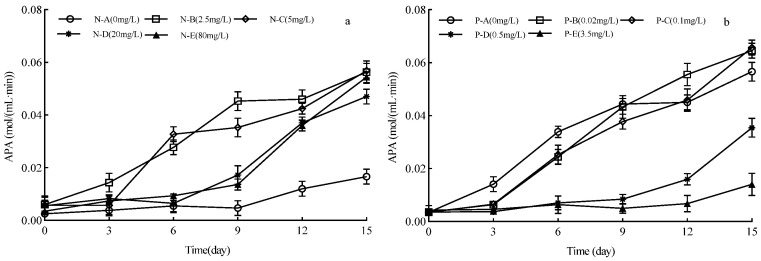
Effects of different N and P concentrations on alkaline phosphatase activity (APA) of *Achnanthidium catenatum*. (**a**) Dynamic changes in APA of *A. catenatum* in different exogenous N concentration groups during the 0–15 day cultivation period; (**b**) dynamic changes in APA of *A. catenatum* in different exogenous P concentration groups during the 0–15 day cultivation period.

During the first 3 days of the experiment (*A. catenatum*’s lag phase), no significant difference in APA was observed among any of the N treatment groups. As the cultures entered the stationary phase, APA gradually increased in each N group, with significant differences among the treatment groups (*p* < 0.05). Low-N treatments (N ≤ 5 mg/L) had significantly higher APA than high-N treatments (N > 5 mg/L) (Figure 5a), and APA was significantly correlated with cell density across all N treatments (*p* < 0.05). In the low-P treatments (P ≤ 0.1 mg/L) and the control, APA increased linearly (Figure 5b)—significantly higher than in the high-P treatments (P > 0.5 mg/L). Additionally, APA showed a significant negative correlation with water P concentration (*p* < 0.05).

### 2.5. Responses of MDA Content in Achnanthidium catenatum to N and P

**Figure 6:**
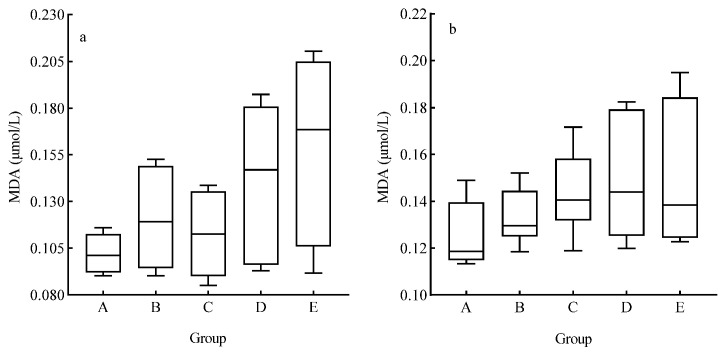
Effects of different N and P concentrations on the average malondialdehyde (MDA) content of *Achnanthidium catenatum*. (**a**) Average MDA content of *Achnanthidium catenatum* in different exogenous N concentration groups; (**b**) average MDA content of *A. catenatum* in different exogenous P concentration groups.

During the experiment, *A. catenatum*’s MDA content was relatively low in all N treatment groups during the lag and log phases, and slightly increased in the stationary phase. The stress resistance of *A. catenatum* to different N concentration treatments varied significantly. The MDA contents in the medium- to low-N treatment groups were relatively low, reflecting milder cell damage and more suitable for its growth; however, the MDA content in the high-N group increased significantly, similar to the changes in the total cell density. These results show that the promoting effect on *A. catenatum* growth weakened at high N concentrations (Figure 6a). In all P treatments, MDA content was relatively low in the lag phase, and gradually increased in the log and stationary phases (Figure 6b), and there was no significant difference in MDA content among the different P treatments (*p* > 0.05).

## 3. Discussion

### 3.1. Effects of N and P on Bloom Formation by Achnanthidium catenatum

Previous studies have shown that diatoms prefer nitrate-rich waters, and increasing nitrate concentration within a certain range promotes their rapid proliferation [20]. This may be due to significantly enhanced nitrate-transport-related enzyme activity in some diatoms under nitrate culture [21]. *A. catenatum* was sensitive to N, its generation time and peak cell density varied with N concentration, and the highest peak density was observed at 2.5 mg/L. These results indicate that the promoting effect on its growth weakened at high N concentrations. Changes in MDA content directly reflect this impact; for instance, the MDA content in the high-N treatment groups increased significantly during the stationary phase. Zheng et al. [22] found that increasing nitrate concentration facilitated *Stephanodiscus* sp. growth within the N concentration range of 0–10 mg/L, whereas ammonia N concentrations exceeding 5 mg/L inhibited its growth. The results of this study indicated that the N response threshold of *A. catenatum* was ≤5 mg/L. Additionally, previous studies have found substantial variability in total N concentrations in water bodies with blooms of *A. catenatum*, ranging from 0.2 to 2.07 mg/L [6]. Similarly, the optimal N concentration for *A. catenatum*’s massive proliferation identified in this study is consistent with this range. A field investigation in Hengshan Reservoir of the Taihu Lake Basin showed that water N influences diatom population density and abundance [23], and Wu et al. [3] confirmed that the increase in N concentration is a critical factor inducing *Stephanodiscus* sp. blooms. The distinct growth traits of *A. catenatum* were short lag phase (3 days), short generation time under low N, and high peak biomass. These findings indicate that *A. catenatum* is N-sensitive, and even relatively low exogenous N input may trigger rapid, low-intensity, short-duration diatom blooming.

Water nutrient analyses from *A. catenatum* bloom events reveal that it is a euryphosphic species, capable of extensive proliferation across a wide P concentration range; the experimental P gradient (0.02–3.5 mg/L) essentially covers most natural water P concentrations. *A. catenatum* was found to be insensitive to P fluctuations and had a relatively long lag phase and short stationary phase. For example, its biomass increased with increasing P, and reached a maximum density of 2.55 × 10^6^ cells/L at 0.1–0.5 mg/L; however, no significant biomass difference occurred at 0.5 and 3.5 mg/L. Although it can adapt to a broad P range, higher P does not lead to significant proliferation. This is consistent with the P nutritional requirements of *Stephanodiscus* sp., a bloom-forming diatom in the Hanjiang River [13]. Physiological indicators further confirm *A. catenatum*’s P response: APA decreased and MDA content increased with rising P. These results show intensified growth stress on the diatom under high P conditions. Notably, *A. catenatum* did not form diatom bloom under indoor low-P conditions (≤0.02 mg/L); however, it blooms in natural lakes and reservoirs in which P concentrations are relatively low (0.01–0.03 mg/L) [6]. It can be observed that the P concentration required for the formation of blooms in indoor settings differs from those at which diatom blooms occur in natural lakes and reservoirs, which may be associated with the nitrogen-to-phosphorus (N/P) ratio of the culture medium. Previous studies show that the N/P ratio is closely linked to massive diatom proliferation: Rhee [24] proposed that high or low N/P ratios (>30 or <5) correspond to P or N limitation, respectively. An N/P ratio <5 at 3.5 mg/L P significantly weakened the growth of *A. catenatum* in this study. Additionally, elevated N or P concentrations can drastically alter the water column N/P ratio; therefore, the phenomenon that both high concentrations of N or P were unsuitable for the growth of *A. catenatum* was observed in the experiment. Unlike N-driven blooms, P-driven *A. catenatum* blooms may progress more slowly, have a shorter duration, and exhibit higher intensity. Therefore, in the prevention and control of *A. catenatum* blooms, local environmental and management authorities should strengthen the monitoring frequency of N and P nutrients in inflow rivers and reservoirs, and pay close attention to monitoring the dynamic changes in the physical and chemical indicators of reservoirs.

### 3.2. Effect of N and P on EPS and Multicellular Chain Colonies

Diatom-secreted EPSs have a crucial effect on their adhesion, movement, cell colony formation, and self-protection [25]. However, EPS secretion and accumulation are influenced by numerous factors, and different nutrients exert distinct effects on EPS secretion by diatoms [26,27]. For example, under N-limited conditions, *Chaetoceros* sp. and *Skeletonema costatum* secreted more EPSs during the log phase than during the stationary phase, and their EPS content increases with rising N within a specific N range [26,28]; however, P exerts a distinct influence on diatom EPS secretion. For instance, *Thalassiosira pseudonana* secreted the highest EPS content under moderate P concentration conditions, which indicated no significant correlation between P concentration and diatom EPS secretion [29]. Comparing the EPS contents across different treatments in this study, *Achnanthidium catenatum* produced EPSs (especially AEPSs) throughout the lag and log phases, but CEPSs were massively synthesized in the stationary phase, with content higher than AEPSs. Similar studies showed that three diatom species synthesized distinct EPS types in the log phase [27], with CEPSs increasing from 20% (log phase) to 30–69% (stationary phase) and peaking in the latter. This study’s findings are consistent with previous observations that diatom EPSs accumulate in the late growth stage [30]. Analyses revealed that the proportion of EPSs produced by *A. catenatum* varies with growth stage during the log and stationary phases, which suggests that CEPSs have an important effect on the dominance of single cells during the stationary phase. Studies on planktonic diatoms demonstrated less CEPS secretion in the log than stationary phase, and diatoms secreted more CEPSs to avoid or mitigate environmental damage to cells under harsher environments [31]. For example, *Navicula pinna* synthesized high EPS levels in the stationary growth phase, which was potentially linked to nitrate deficiency, while appropriate N limitation enhance EPS production [32]. The EPS secretion pattern of *A. catenatum* in this study was similar to that reported by Staats et al. [26]. AEPSs of *Cylindrotheca closterium* were produced throughout growth, while CEPSs were massively produced in the stationary phase; however, *A. catenatum* secreted comparable amounts of AEPSs and CEPSs during the log phase, possibly due to different EPS secretion mechanisms among different types of diatoms.

Previous studies have shown a significant positive correlation between EPS content and cell density in *Nitzschia perminuta* [27]. In this experiment, under high N and P conditions, *A. catenatum* synthesized more CEPSs than AEPSs in the stationary phase, and the CEPS content increased with cell density. This phenomenon may be associated with dynamic shifts in the unicellular and multicellular chain colony ratio during *A. catenatum*’s growth cycle. Small unicellular diatom cells exhibited low resistance to environmental stress and were more susceptible to predation by filter-feeding fish and zooplankton [33]; in contrast, the formation of multicellular chain colonies moderately enhances their defensive capabilities. The density of chain colonies was correlated with AEPS content, suggesting that AEPSs were involved in the formation of multicellular chain colonies. *A. catenatum* tended to adopt a unicellular lifestyle during the stationary growth phase. This could be attributed to the lack of competitive pressure from other organisms under indoor culture conditions. Moreover, growth in the stationary phase was constrained by nutrient limitation, which caused the density of multicellular chain colonies to decline and unicellular individuals to reach peak density. As a result, the CEPS content also reached its maximum level during the stationary phase. Additionally, other studies have shown that in environments with stable nutrient concentrations, unicellular or small-colony *Microcystis* utilizes nutrients and light more efficiently [34]. The potential instability of the reported EPS component dynamics constitutes a limitation of this study as it may undermine the reliability to interpret the formation of multicellular chain colonies and unicellular dominance at different growth stages. In subsequent studies, the separation protocol for AEPSs and CEPSs should be further optimized and validated to reduce the risk of cross-contamination and improve the accuracy of EPS fractionation.

In conclusion, *A. catenatum* can grow rapidly and increase the proportion of unicellular individuals under low N and P conditions, which is conducive to its spread and migration in aquatic environments. This may further expand the scale of diatom blooms and is also one of the main reasons why *Achnanthes lanceolata* has become a typical dominant bloom-forming diatom; therefore, to prevent the outbreak of *A. catenatum* blooms, it is necessary to strengthen the monitoring and regulation of N and P nutrient levels in lake and reservoir waters.

## 4. Materials and Methods

### 4.1. Experimental Materials

Log-phase *Achnanthidium catenatum* culture was aliquoted into two 10 mL fractions, transferred to centrifuge tubes (NEST Biotechnology Co., Ltd., Wuxi, China), and centrifuged at 1968× *g* (Hunan Xiangyi Laboratory Instrument Development Co., Ltd., Changsha, China) for 10 min. After removing the supernatant, the precipitated cell was inoculated respectively into nitrogen-and phosphorus-free media and subjected to 5-day nutrient starvation (N and P depletion) for subsequent experiments.

### 4.2. Experimental Design

Based on a nitrogen- and phosphorus-free CSI medium [35], two experiments were conducted in this study to establish concentration gradients for N and P, respectively. N treatments: 0 (control, N-A), 2.5 (low N, N-B), 5 (medium N, N-C), 20 (high N, N-D), and 80 mg/L (high N, N-E); and P treatments: 0 (control, P-A), 0.02 (low P, P-B), 0.1 (medium P, P-C), 0.5 (high P, P-D) and 3.5 mg/L (high P, P-E). After starvation, *Achnanthidium catenatum* cells were inoculated into respective treatment media (150 mL per replicate, 3 replicates per group), cultured in a light incubator (Shaoguan Taihong Medical Equipment Co., Ltd., Shaoguan, China), and shaken every 4 h under the conditions of 23 ± 1 °C, 50 μmol/(m^2^·s), and a 12:12 h light:dark cycle. In this experiment, the N and P concentration gradients basically covered the N and P levels during *A. catenatum* blooms in reservoir and lake waters [9,10,11], and the gradients were determined through preliminary experiments.

### 4.3. Determination of Growth and Physiological Indices of Achnanthidium catenatum

#### 4.3.1. Cell Density

The initial density of *A. catenatum* was determined, with subsequent measurements taken at 3-day intervals during the experiment. When counting under an optical microscope (Olympus, Tokyo, Japan), the densities of unicellular individuals and multicellular chain colonies (≥2 valve-connected cells) were recorded separately. Total cell density was calculated as the sum of these two components.

#### 4.3.2. Determination of EPS Content

AEPSs and CEPSs were quantified at 3-day intervals via the phenol–sulfuric acid method (phenol and concentrated sulfuric acid; Sinopharm Chemical Reagent Co., Ltd., Beijing, China) [36], with total EPS as their sum. For each treatment, 10 mL of *A. catenatum* culture was centrifuged at 12,879× *g* for 15 min at 4 °C. The supernatant was filtered through a 0.45 μm membrane (Tianjin Jinteng Experimental Equipment Co., Ltd, Tianjin, China) filter to obtain CEPSs. The pellet was resuspended in sterile distilled water, heated in a water bath (Changzhou Aohua Instrument Co., Ltd., Changzhou, China) at 60 °C for 30 min, and centrifuged again for 15 min. The resulting supernatant was filtered through a 0.22 μm membrane (Tianjin Jinteng Experimental Equipment Co., Ltd, Tianjin, China) filter to collect AEPSs. Both polysaccharide solutions were dialyzed for 48 h using dialysis bags (Beijing Solarbio Science & Technology Co., Ltd., Beijing, China), and the dialysates’ absorbance was measured at 490 nm (Shimadzu, Kyoto, Japan).

#### 4.3.3. Determination of APA

Every 3 days, 2 mL of culture from each treatment was transferred to a 10 mL centrifuge tube. Next, 2 mL of 0.05 mol/L Tris–HCl buffer (pH 8.5; Sangon Biotech, Shanghai, China) and 0.2 mL of 10 mmol/L p-nitrophenyl phosphate disodium salt (pNPP; Sinopharm Chemical Reagent Co., Ltd., Shanghai, China) substrate were added. After 3 h of incubation at 37 °C, 1 mL of 0.1 mol/L NaOH (Sinopharm Chemical Reagent Co., Ltd., Beijing, China) was added, and the mixture was vortexed thoroughly (5.Shanghai Huyuemeng Scientific Instrument Co., Ltd., Shanghai, China). Following centrifugation, the supernatant was collected and its absorbance measured at 405 nm.

#### 4.3.4. Determination of MAD

In accordance with the method outlined by Du and Bramlage [37], 5 mL of *A. catenatum* culture from each treatment was collected every 3 days. The samples were centrifuged at 1968× *g* for 5 min, and the supernatant discarded. After adding 50 mmol/L phosphate buffer (4.Sangon Biotech, Shanghai, China), the mixture was ground into a powder with liquid N. Following 10 min of centrifugation, 2 mL of the supernatant was mixed with 2.5 mL of 0.5% thiobarbituric acid (TBA; Sinopharm Chemical Reagent Co., Ltd., Shanghai, China) solution, incubated in a boiling water bath for 30 min, cooled to room temperature, and recentrifuged. The resulting supernatants were collected, and their absorbance was measured at 450, 532, and 600 nm.

### 4.4. Statistical Analysis

Microsoft Excel 2010 was used for data calculation and processing, and SPSS 21.0 for correlation and significance difference analysis.

## 5. Conclusions

(1) N and P exerted divergent effects on the growth of *Achnanthidium catenatum*. Specifically, the N and P concentration thresholds required for the diatom to reach peak bloom density were 2.5 and 0.5 mg/L, respectively. *A. catenatum* was more sensitive to changes in N than in P. This sensitivity may lead to the earlier occurrence and longer duration of algal blooms under N loading, whereas P-driven blooms tend to have a shorter duration but higher intensity.

(2) N and P had significant effects on the formation of multicellular chain colonies of *A. catenatum* (*p* < 0.05). The diatom tended to adopt a unicellular lifestyle during the stationary phase, and this characteristic might further promote the expansion of algal bloom coverage. 

(3) The EPS content in *A. catenatum* varied with its growth stage and was highly correlated with the proportion of its unicellular and multicellular chain colony lifestyles.

## Data Availability

The datasets analyzed during the current study are available from the corresponding author on reasonable request.
